# Factors associated with overall survival in breast cancer patients with leptomeningeal disease (LMD): a single institutional retrospective review

**DOI:** 10.1186/s13058-024-01789-7

**Published:** 2024-03-29

**Authors:** Gerald Wallace, Ronak Kundalia, Ethan Vallebuona, Biwei Cao, Youngchul Kim, Peter Forsyth, Aixa Soyano, Inna Smalley, Yolanda Pina

**Affiliations:** 1 Department of Neuro-Oncology, H. Lee Moffitt Cancer Center and Research Institute, 12902 USF Magnolia Dr., Tampa, FL 33612 USA; 2 Department of Neurology, Medical College of Georgia, Augusta, GA USA; 3 Department of Metabolism and Physiology, H. Lee Moffitt Cancer Center and Research Institute, Tampa, FL USA; 4 Department of Biostatistics and Bioinformatics, H. Lee Moffitt Cancer Center and Research Institute, 12902 USF Magnolia Dr., Tampa, FL 33612 USA; 5 Department of Tumor Biology, H. Lee Moffitt Cancer Center and Research Institute, Tampa, FL USA; 6 Department of Breast Oncology, H. Lee Moffitt Cancer Center and Research Institute, Tampa, FL USA

## Abstract

**Background:**

Breast cancer-related leptomeningeal disease (BC-LMD) is a dire diagnosis for 5–8% of patients with breast cancer (BC). We conducted a retrospective review of BC-LMD patients diagnosed at Moffitt Cancer Center from 2011 to 2020, to determine the changing incidence of BC-LMD, factors which are associated with the progression of BC CNS metastasis to BC-LMD, and factors which are associated with OS for patients with BC-LMD.

**Methods:**

Patients with BC and brain/spinal metastatic disease were identified. For those who eventually developed BC-LMD, we used Kaplan–Meier survival curve, log-rank test, univariable, and multivariate Cox proportional hazards regression model to identify factors affecting time from CNS metastasis to BC-LMD and OS.

**Results:**

128 cases of BC-LMD were identified. The proportion of BC-LMD to total BC patients was higher between 2016 and 2020 when compared to 2011–2015. Patients with HR+ or HER2 + BC experienced longer times between CNS metastasis and LMD than patients with triple-negative breast cancer (TNBC). Systemic therapy and whole-brain radiation therapy (WBRT) was associated with prolonged progression to LMD in all patients. Hormone therapy in patients with HR + BC were associated with a delayed BC-CNS metastasis to LMD progression. Lapatinib treatment was associated with a delayed progression to LMD in patients with HER2 + BC. Patients with TNBC-LMD had shorter OS compared to those with HR + and HER2 + BC-LMD. Systemic therapy, intrathecal (IT) therapy, and WBRT was associated with prolonged survival for all patients. Lapatinib and trastuzumab therapy was associated with improved OS in patients with HER2 + BC-LMD.

**Conclusions:**

Increasing rates of BC-LMD provide treatment challenges and opportunities for clinical trials. Prospective trials testing lapatinib and/or similar tyrosine kinase inhibitors, IT therapies, and combination treatments are urgently needed.

**Supplementary Information:**

The online version contains supplementary material available at 10.1186/s13058-024-01789-7.

## Introduction

Leptomeningeal disease (LMD) is a dreadful complication occurring in approximately 5–8% of patients with breast cancer (BC) [1]. Median overall survival (OS) in untreated patients with LMD including from BC is approximately 1 month [1, 2]. Aggressive treatment in breast cancer-related leptomeningeal disease (BC-LMD) can extend OS to 3–4 months, although this is BC subtype-specific [3, 4]. Beyond receptor subtype stratification and examining the efficacy of HER2-targeted therapy, only a few studies have attempted to identify the clinical characteristics of BC-LMD [5–7].

An earlier diagnosis of LMD is important to improve patient survival and enrollment in clinical trials. However, Magnetic Resonance Imaging (MRI) has a wide variability to accurately diagnose LMD [8], and the current gold standard of a positive CSF cytology has a very low sensitivity (< 50%) and positive predictive value [9]. To optimize the diagnostic yield beyond traditional CSF cytology, assays that detect circulating tumor cells and cell-free DNA have been developed over the past decade, but the clinical use of these new techniques to diagnose and treat LMD has not yet been defined [10].

Breast cancer in the central nervous system (BC CNS) has been under-recognized, with the real epidemiological data likely under-reported, especially for those who develop BC-LMD [11]. A concerted effort to identify and treat BC-LMD at Moffitt Cancer Center (MCC) began around 2015 and is currently ongoing. This study aims to determine if the incidence of BC-LMD at MCC is changing over time, what factors may impact progression of BC CNS metastasis to LMD, and what factors may affect the OS for patients with BC-LMD.

## Methods

This project was approved by the Scientific Review Committee and Institutional Review Board at MCC (MCC #21,524). A retrospective review of medical records was conducted to identify patients diagnosed and/or treated at MCC with BC and who also had a diagnosis of CNS metastases between January 1, 2011 and December 31, 2020. Only patients with confirmed diagnosis of BC and LMD were included. LMD diagnosis was confirmed with CSF cytology and/or MRI. Patients were excluded if the LMD diagnosis was based solely on clinical suspicion, if confirmatory MRI or CSF cytology was not available in the medical record, or if there was another malignancy which might seed the leptomeningeal space.

### Data collection

Demographics, BC receptor subtype, dates of BC/CNS metastasis/LMD diagnoses, and dates of censorship/death were collected. Method of diagnosis, treatments prior to and following CNS metastasis, and treatments following BC-LMD were also collected.

### Statistical analysis

Descriptive statistics including frequency, percentage, median, and range were calculated for patients’ demographics and clinical characteristics. Differences in continuous variables between patient groups were statistically tested using the Kruskal–Wallis tests. The associations between categorical variables and endpoints were evaluated using Chi-square test or Fisher's exact test.

Median time between CNS metastatic disease and LMD, and OS post-LMD were estimated using the Kaplan–Meier method. Univariable Cox proportional hazards regression analysis was used to estimate hazard ratios and their 95% confidence interval (CI). Significant variables at univariable analysis were subsequently tested in multivariable Cox regression analysis. Univariable analysis comparing patients who received a therapy versus those who did not receive the same therapy includes all patients who received this therapy, some of whom may have received more than one therapy. All reported *p* values were two—sided, and significance level was 0.05 (*p* < 0.05). Analyses were performed using R version 4.1.0.

## Results

### Cases and Demographics

**Fig. 1:**
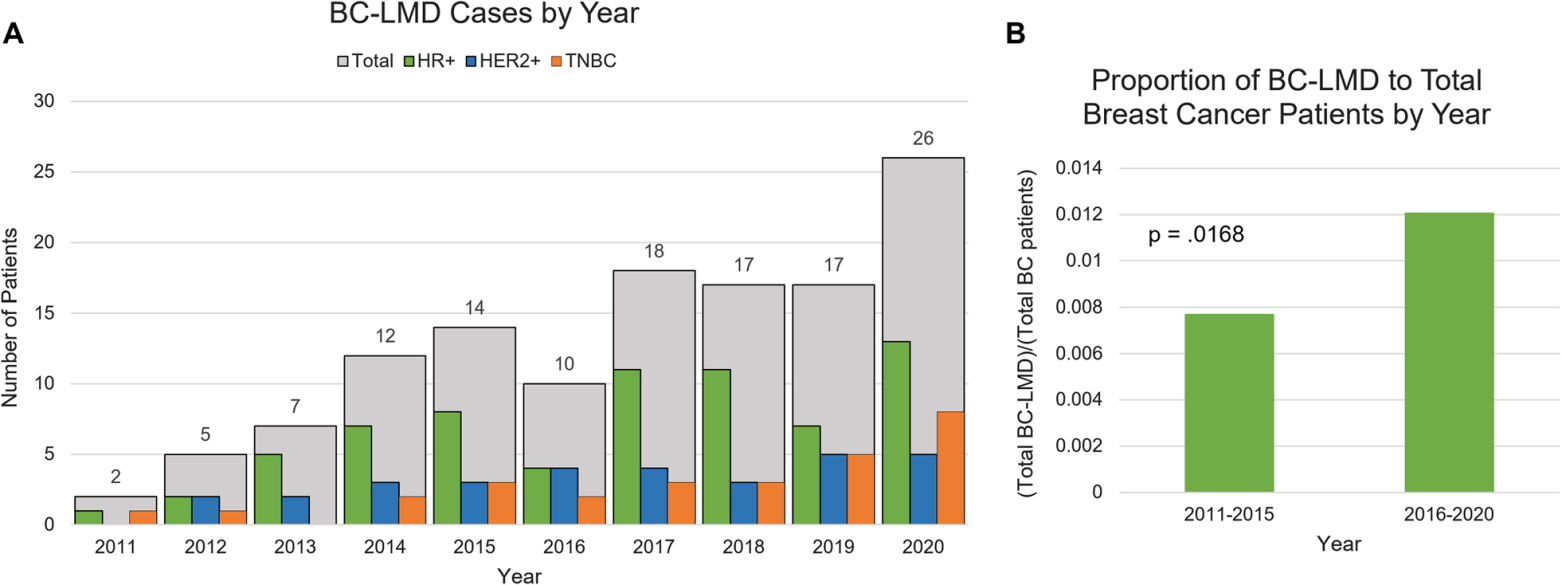
**A** Total number of BC-LMD cases seen at Moffitt Cancer Center between 2011 and 2020, by breast cancer subtype. **B** The proportion of BC-LMD patients to total breast cancer patients seen at Moffitt Cancer Center between 2011 and 2015 and 2016–2020. A significantly higher proportion of (BC-LMD patients)/(Total Breast Cancer Patients) was observed in the latter half of the decade (*p* = 0.0168). *HR* + Hormone Receptor Positive; *HER2* + Human Epidermal Growth Factor Receptor 2-Positive; *TNBC* Triple Negative Breast Cancer; *BC-LMD* Breast Cancer Leptomeningeal Disease

One-hundred-twenty-eight patients were identified who met radiographic and/or CSF cytology criteria for BC-LMD diagnosis. Forty patients were identified between 2011 and 2015, whereas 88 patients were identified between 2016 and 2020 (Fig. 1A). The proportion of BC-LMD patients to total BC patients was significantly higher between 2016 and 2020 when compared to 2011–2015 (*p* = 0.0168, Fig. 1B). The median age of BC diagnosis was 51 years [22–79 years]. The median age of CNS metastasis diagnosis was 53 years [26–81 years] and median age of LMD diagnosis was 54 years [27–83 years].

Of the 128 patients with BC-LMD, 66 had HR + BC (52%), 34 had HER2 + BC (26%), and 28 had TNBC BC (22%). Median age at BC diagnosis significantly differed between all three groups (HR+: 55 years, [25–76]; HER2+: 50.5 years, [22–79]; TNBC: 45.5 years, [24–69]; *p* = 0.0348). The median age at LMD diagnosis between disease subtypes significantly differed (HR+: 58.5 years, [27–79]; HER2 + : 53.5 years, [29–83]; TNBC: 48 years, [27–70]; *p* = 0.0023).

Out of all the patients identified, 100 (78%) had systemic metastasis prior to the diagnosis of LMD, and 114 (89%) had CNS metastasis prior to the diagnosis of LMD. Patients with HR+ and HER2 + BC were more likely to develop systemic metastasis prior to the diagnosis of LMD when compared to patients with TNBC (i.e., 86% and 79%, respectively, versus 57%; *p* = 0.007). All three patient cohorts were equally likely to develop CNS metastatic disease prior to the diagnosis of LMD. Patients’ demographic data is summarized in Table 1.

**Table 1:** Demographic data for all patients diagnosed with BC-LMD at Moffitt Cancer Center between 2011 and 2020

	Total	HR +	HER2 +	TNBC
Number of patients	128	66	34	28
Median age at BC diagnosis	51 [22, 79]	55 [25, 76]	50.5 [22, 79]	45.5 [24, 69]
Median age at CNS metastasis	53 [26, 81]	55 [38, 68]	52 [32, 81]	47 [26, 66]
Median age at LMD diagnosis	54 [27, 83]	58.5 [27, 79]	53.5 [29, 83]	48 [27, 70]
Race				
Black	12 (9.3%)	7 (11%)	1 (2.9%)	4 (14%)
Other	21 (16%)	10 (15%)	6 (18%)	5 (18%)
White	95 (74%)	49 (74%)	27 (79%)	19 (68%)
Stage at BC diagnosis				
<=2	43 (34%)	21 (32%)	10 (29%)	12 (43%)
=>3	77 (60%)	41 (62%)	22 (65%)	14 (50%)
Systemic metastasis	100 (78%)	57 (86%)	27 (79%)	16 (57%)
CNS metastasis	114 (89%)	56 (85%)	31 (91%)	27 (96%)
MRI only confirmed LMD diagnosis	70 (55%)	40 (61%)	19 (56%)	11 (39%)
MRI + CSF confirmed LMD diagnosis	54 (42%)	25 (38%)	14 (41%)	15 (54%)

*BC* Breast Cancer; *HR* + Hormone Receptor Positive; *HER2* + Human Epidermal Growth Factor Receptor 2-Positive; *TNBC* Triple Negative Breast Cancer; *CNS* Central Nervous System; *LMD* Leptomeningeal Disease; *MRI* Magnetic Resonance Imagining; *CSF* Cerebrospinal Fluid

### Factors affecting time between CNS metastasis diagnosis and LMD diagnosis

Only patients who had at least a one-month gap between CNS metastasis and LMD diagnoses were included in this analysis. This stratification ensured that only patients with discrete non-LMD CNS metastasis diagnosis and LMD diagnosis were analyzed in this comparison. Of the 48 BC-LMD patients that met this criterion, 19 had HR + BC (40%), 19 HER2 + BC (40%), and 10 TNBC (20%). Demographics for these patients is summarized in Table 2, and post-CNS metastasis/pre-LMD treatment data is summarized in Additional file 1: Table S1.

**Table 2:** Demographic data for patients with at least one month between the CNS metastatic disease diagnosis and the BC-LMD diagnosis

	Total	HR +	HER2 +	TNBC
Number of patients	48	19	19	10
Median age at CNS metastasis	50.5 [26, 81]	51 [38, 68]	52 [32, 81]	47.5 [26, 66]
Median age at LMD diagnosis	51.5 [27, 82]	52 [39, 70]	53 [37, 82]	48 [27, 68]
Race				
Black	3 (6%)	1 (5.3%)	1 (5.3%)	1 (10%)
Other	5 (10%)	3 (16%)	2 (11%)	0 (0%)
White	40 (83%)	15 (79%)	16 (84%)	9 (90%)
Stage at diagnosis				
<=2	17 (35%)	6 (32%)	6 (35%)	5 (50%)
=>3	29 (60%)	13 (68%)	11 (65%)	5 (50%)
MRI only confirmed LMD diagnosis	28 (58%)	14 (74%)	10 (53%)	4 (40%)
MRI + CSF confirmed LMD diagnosis	20 (42%)	5 (26%)	8 (42%)	6 (60%)

*BC* Breast Cancer; *HR* + Hormone Receptor Positive; *HER2* + Human Epidermal Growth Factor Receptor 2-Positive; *TNBC* Triple Negative Breast Cancer; *CNS* Central Nervous System; *LMD* Leptomeningeal Disease; *MRI* Magnetic Resonance Imagining; *CSF* Cerebrospinal Fluid

**Fig. 2:**
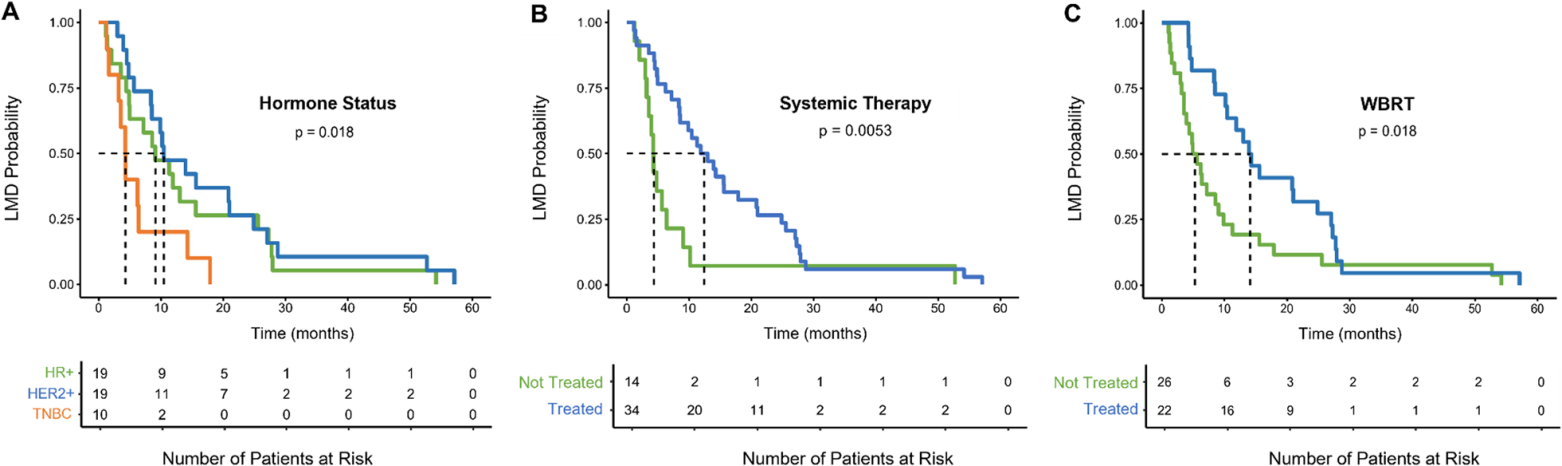
Kaplan–Meier estimates for factors affecting time between CNS-metastasis diagnosis and BC-LMD diagnosis. **A** Patients with TNBC experienced a significantly shorter median time between breast cancer CNS-metastasis diagnosis and BC-LMD (4.3 months) compared to HR + (9.1 months) and HER2 + (10.5 months) patients. **B** Patients that received systemic therapy experienced a longer median time between CNS metastasis and BC-LMD (12.5 months) than patients who did not receive any systemic therapy (4.3 months). **C** Patients that received WBRT had a longer median time between their CNS metastasis and BC-LMD diagnosis (14.1 months) compared to patients that did not receive WBRT (5.3 months). *HR* + Hormone Receptor Positive; *HER2* + Human Epidermal Growth Factor Receptor 2-Positive; *TNBC* Triple Negative Breast Cancer; *LMD* Leptomeningeal Disease; *WBRT* Whole Brain Radiation Therapy

Patients with HR + BC and HER2 + BC experienced longer time between BC-CNS metastasis to BC-LMD diagnosis compared to patients with TNBC disease (Fig. 2A, *p* = 0.018). Patients who received systemic treatments post-CNS metastasis experienced a longer time to LMD diagnosis (12.5 months [8.6, 20.8)] compared to patients who did not receive systemic therapy (4.3 months [3.6, 10.2]; Fig. 2B, *p* = 0.0053). Furthermore, patients who received WBRT post-CNS metastasis diagnosis had a median time to LMD diagnosis of 14.1 months [10.5, 27.1], while patients who did not receive WBRT had a median time of only 5.3 months [3.6, 9.9] between their CNS metastasis and LMD diagnoses (Fig. 2C, *p* = 0.018).

Patients with HR + BC who received any hormone therapy had significantly longer times between their CNS metastasis diagnosis and LMD diagnosis (Additional file 2: Fig. S1A, *p* = 0.0005). More specifically, HR + patients that were treated with letrozole had a longer time to LMD diagnosis from CNS metastasis diagnosis (Additional file 2: Fig. S1B, *p* = 0.0007). Similar trends were observed in HR + BC patients who were treated with the aromatase inhibitor exemestane (Additional file 2: Fig. S1C, *p* = 0.0015) and CDK4/6 inhibitor palbociclib (Additional file 2: Fig. S1D, *p* = 0.023). Finally, the patients treated with mTOR inhibitor everolimus also experienced prolonged time between CNS metastasis and LMD diagnosis in HR + BC patients (Additional file 2: Fig. S1E, *p* = 0.0013). Of all these treatments, exemestane and palbociclib also showed significance in multivariate analysis (Additional file 2: Fig. S1F).

Anti-HER2 therapy lapatinib was associated with longer times between CNS metastasis and BC-LMD (Additional file 2: Fig. S2, *p* = 0.026) for HER2 + BC-LMD patients (n = 10) when compared to patients of any subtype who did not receive this treatment. Unfortunately, no systemic therapy was found to be associated with significantly longer time between CNS metastasis diagnosis to LMD diagnosis in TNBC patients.

Finally, in patients with a prior history of either brain tumor excision (n = 21) and radiosurgery (n = 32), no differences in the time between CNS metastasis and LMD was observed when compared to patients who did not receive such treatments (Additional file 2: Fig. S3).

### OS with BC-LMD

**Fig. 3:**
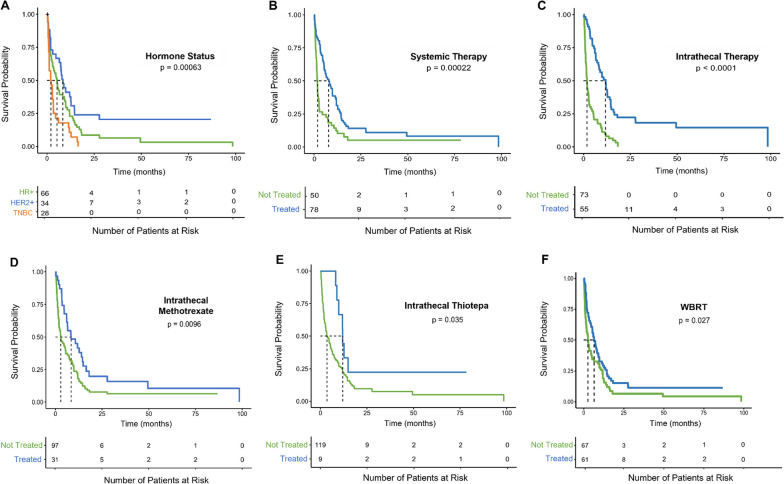
Kaplan–Meier estimate for factors affecting overall survival in BC-LMD patients. **A** TNBC-LMD patients had a significantly lower overall survival (2 months) when compared to HR + BC-LMD patients (5.3 months) and HER2 + BC-LMD patients (8.4 months). Median survival time between HR + and HER2 + BC-LMD patients did not significantly differ. **B** Patients receiving systemic therapy post BC-LMD diagnosis had a significantly higher median survival time (7.9 months) when compared to BC-LMD patients that did not receive systemic therapy (1.8 months). **C** Patients receiving intrathecal therapy post BC-LMD diagnosis had a significantly higher median survival time (11.8 months) when compared to BC-LMD patients that did not receive intrathecal therapy (1.9 months). **D, E** More specifically, overall survival median times were higher in patients that received intrathecal methotrexate (8.4 months) and/or intrathecal thiotepa (12 months) versus those that did not (2.9 months; 3.6 months, respectively). **F** patients receiving WBRT post BC-LMD diagnosis had a higher overall survival median time (6.5 months) than those who did not receive WBRT (2.7 months). *HR* + Hormone Receptor Positive; *HER2* + Human Epidermal Growth Factor Receptor 2-Positive; *TNBC* Triple Negative Breast Cancer; *LMD* Leptomeningeal Disease; *WBRT* Whole Brain Radiation Therapy; *IT* Intrathecal; *HR* Hazard Ratio; *CI* Confidence Interval

Treatment data for patients post-LMD diagnosis is summarized in Additional file 1: Table S2. Patients with TNBC had significantly worse median post-LMD OS (2 months, [1.1, 3.4]), when compared to both patients with HER2 + (8.4 months [6.5, 14.5]; *p* = 0.0016) and HR + BC (5.3 months, [2.9, 9.3]; *p* = 0.0097). Survivability by cancer subtype is summarized in Fig. 3A.

**Fig. 4:**
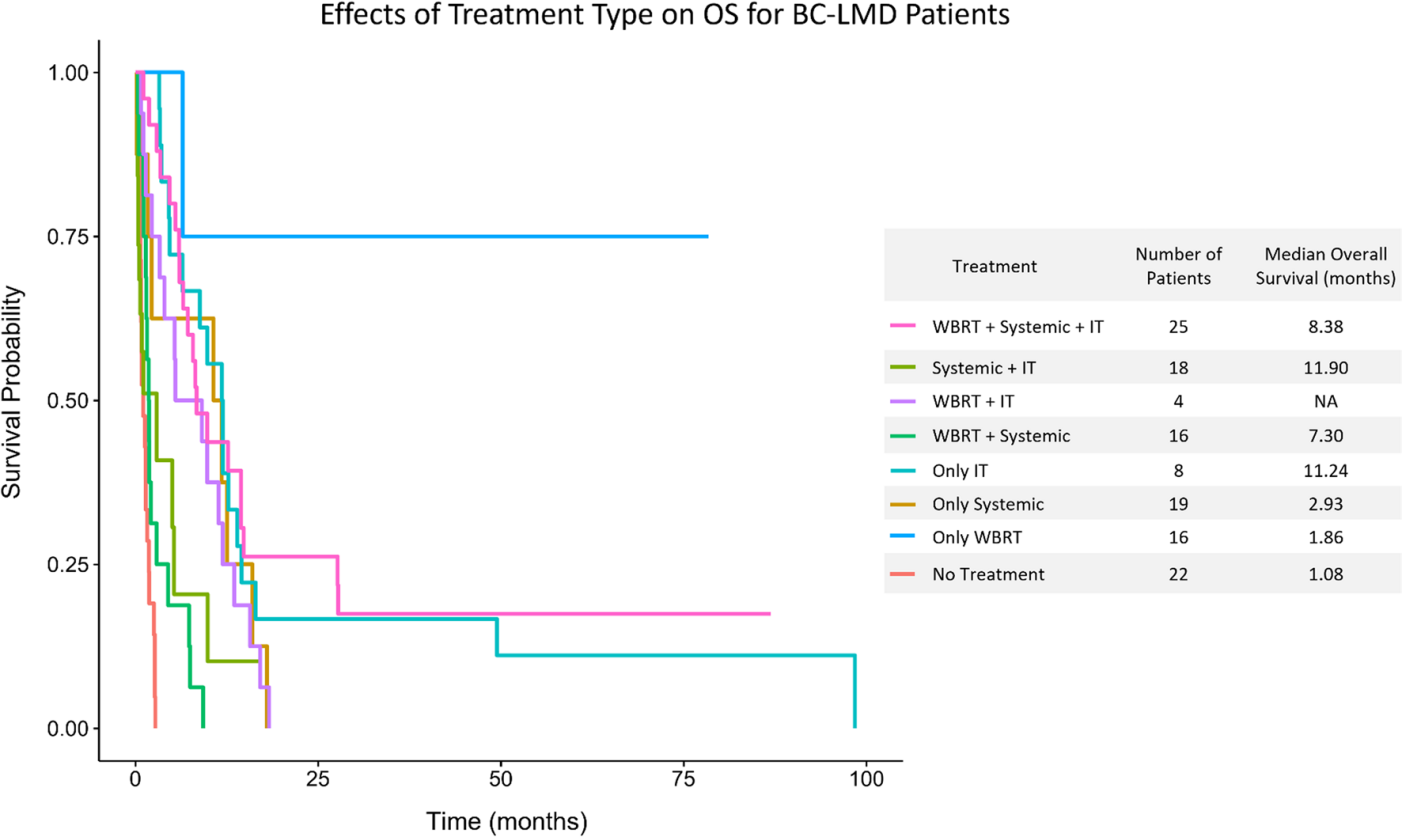
Kaplan Meier analysis of BC-LMD patients receiving different combinations of WBRT, Systemic Therapy, and/or IT therapy. Patients receiving only IT, systemic + IT, WBRT + IT, WBRT + systemic therapy, or all three therapies had a significantly longer median OS than patients that received no therapy (*p* < 0.05, respectively). Compared to WBRT alone, patients receiving WBRT + systemic therapy or WBRT + systemic therapy + IT therapy had a significantly longer median OS (*p* < 0.0001, respectively). *BC-LMD* Breast Cancer Leptomeningeal Disease; *OS* Overall Survival; *IT* Intrathecal Therapy; *WBRT* Whole Brain Radiation Therapy

22 patients did not receive any treatment post-LMD diagnosis, and they had a median OS of 1.08 months [0.26, 3.00]. Conversely, 106 patients received either systemic treatment, IT therapy, WBRT, or some combination of the three post BC-LMD diagnosis, and their median OS was significantly higher at 6.54 months [0.21, 106.96] (*p* < 0.0001). Median OS by treatment type is summarized in Fig. 4.

Fifty patients did not receive any systemic therapy post-LMD diagnosis, and they had a median OS of 1.8 months [1.4, 2.7]. This includes patients who received no therapy and those who only received IT therapy and/or WBRT. Conversely, 78 patients received systemic therapy post-LMD (alone or in combination with IT therapy and/or WBRT) and they had a significantly improved median OS of 7.9 months [5.5, 11.8] (Fig. 3B, *p* = 0.0002). However, 19 patients received only systemic therapy post LMD diagnosis. Their median OS of 2.93 month was not significantly different than patients who did not receive any treatment post-LMD (Fig. 4).

Furthermore, 55 patients received IT therapy post-LMD diagnosis, and they had a median OS of 11.8 months [8.2, 14.5]. This was significantly greater than the 1.9 month [1.4, 2.7] month median OS of patients that did not receive IT therapy (Fig. 3C, *p* < 0.0001). Specifically, patients receiving IT methotrexate had a 5.5 month longer median OS than patients who did not (Fig. 3D, *p* = 0.0096), and patients receiving IT thiotepa had a median OS that was 8.4 months longer than those who did not (Fig. 3E, *p* = 0.0354). Improved OS was also observed in the 8 patients who received only IT therapy for treatment post LMD diagnosis. Their median OS of 11.24 [0.21, 19.27] months was significantly greater than the OS of the 22 patients that did not receive any therapy post BC-LMD diagnosis (Fig. 4, *p* = 0.041).

Finally, 61 patients receiving WBRT survived a median of 6.5 [4.7, 9.8] months while 67 patients who did not receive WBRT survived a median of 2.7 months [1.7, 5.3] (Fig. 3F, *p* = 0.0274). However, the OS for patients that received only WBRT post-LMD diagnosis was 1.86 months [0.39, 10.11], which was not significantly greater than the OS for patients that received no therapy (Fig. 4).

In the context of HR + BC-LMD, 8 HR + patients treated with capecitabine post BC-LMD diagnosis showed median survival of 14.8 [5.5, N.E.] months while HR + patients that did not receive capecitabine showed a median survival of 4.0 [2.3, 8.2] months. However, this difference was not statistically significant (*p* = 0.0694). Similarly, 11 HR + BC-LMD patients treated with anthracycline showed a median OS of 12.8 (11.8, N.E.) months, which was not statistically longer than the median OS of HR + BC-LMD patients who did not receive anthracycline treatment (3.6 [2.2, 6.4] months, *p* = 0.0661). These marginally significant results are possibly attributable to a low power with small sample size. As noted in the collective patient survival data, IT therapy, and more specifically IT methotrexate, significantly improved OS in patients with HR + BC-LMD when compared to patients that did not receive IT therapy/IT methotrexate (Additional file 2: Fig. S4A/B *p* = 0.0019 and 0.0028, respectively).

For patients with HER2 + BC, systemic treatment in general, particularly anti-HER2 targeted therapies such as trastuzumab and lapatinib, was associated with significantly improved patient median survival (Additional file 2: Fig. S5A–C, *p* = 0.015, 0.0261 and 0.0352, respectively). HER2 + BC-LMD patients receiving IT therapy had an improved median OS by 11.1 months when compared to HER2 + BC-LMD patients that did not receive IT therapy (Additional file 2: Fig. S5D, *p* < 0.0001). Importantly, all HER2 + patients that received IT therapy received IT trastuzumab as a part of their treatment regimen.

Although treatment with systemic therapy collectively was associated with enhanced survival in patients with TNBC when compared to those patients who did not receive systemic therapy (3.4 months vs. 1.2 months, Additional file 2: Fig. S6A, *p* = 0.0334), no individual therapy was found to be associated with prolonged survival in this subset of patients. However, IT therapy was found to be associated with increased OS in TNBC patients compared those that did not receive IT therapy (8.9 months vs. 1.1 months, Additional file 2: Fig. S6B, *p* = 0.001).

## Discussion

Data from MCC supports a significant rise in the number of BC-LMD cases between 2011 and 2020. Greater institutional efforts to identify and treat LMD patients were made in the latter half of the study period, which may have driven the increased in BC-LMD diagnosis. A slight trend towards increased proportions of patients with TNBC were seen in the current review, but all cases increased over the study period. We could not rule out the possibility that there is an overall increase LMD incidence due to improvements in the control of systemic disease, allowing patients to survive longer and allowing more time for CNS metastases to develop. A previous study showed that HR status influences the risk of developing LMD [12]. This is supported by recent data suggesting HR + BC-LMD likely comprises the majority of all cases (range 48–66%), and rates of HER2 + and TNBC-LMD are more variable [12–16]. HER2 + BC-LMD represents 14–47.4% of all cases, and TNBC-LMD varies between 13.1 and 40% across studies [12–16]. The proportion of HR + patients was similar in our study at 51% compared to HER2 + BC-LMD (27%) and TNBC (22%).

The median age of BC diagnosis in the current study of 51 years [22–79 years] is lower than the average reported with a disease peak around age 60 [17, 18]. Although the median age at diagnosis of BC appears to vary across nations, and a similar median age has been reported in other regions across the globe [19]. Data has suggested young age being a poor prognostic factor in BC and associated with more aggressive presentations at diagnosis of metastatic BC [20]. Our current data may support this and may reflect an age shift to a younger population with a predisposition to develop LMD. Future prospective studies may help elucidate this. BC subtype was associated with the time between CNS metastasis and BC-LMD diagnoses. TNBC had the fastest progression to BC-LMD compared to HR + and HER2 + BC-LMD. Even though HER2 + cancer may have a predisposition for CNS invasion, a tendency to invade the leptomeninges has not been clinically revealed [1, 21]. It has been shown that increased survival in patients with BC generally correlates with increased incidence of CNS metastases [22]. Prolonged survival and risk of developing metastases, may confer a greater risk of developing BC-LMD [14]. Historically, HER2 + and TNBC have carried an increased risk of developing CNS metastasis, and an associated decreased survival relative to HR + disease [23, 24]. Earlier work suggested no difference among BC-LMD based on the molecular subtype [25]. However, our findings support more recent studies demonstrating an increased risk of developing LMD for TNBC [12, 13, 15, 16, 26].

This study also suggests that the use of any systemic treatment and/or WBRT post-CNS metastasis may delay progression from to BC-LMD. HR + patients receiving hormone therapies and/or kinase inhibitors may experience prolonged times between CNS metastasis and LMD diagnosis. For HER2 + patients, treatment with lapatinib demonstrated similar results. However, the studies stringent criterion for stratifying patients by at least a one-month period between CNS metastasis and LMD severely reduced the total patient sample from 128 to 48 patients. Factors shown to contribute to the development of LMD from CNS metastasis include genomic alterations independent of primary tumor site in MAPK, CDH2, and SF3BI displaying significant gain-, loss- and switch-of-function mutations, respectively [27].

Due to the exploratory, retrospective nature of our study and the limited number of patients from whom data is available for analysis, we can only begin to examine the associations between treatment variables and patient outcomes. In many cases, patients received a combination of therapies, potentially confounding the associations or individual therapies with outcome. Follow-up, prospective studies analyzing significantly larger patient samples and basic science investigations in animal models of LMD are required to definitively determine factors that directly influence time between CNS metastasis and BC-LMD. For example, we found that patients with HER2 + BC and LMD who received any hormone therapy had significantly longer times between their CNS metastases and LMD diagnosis, however, this could be due to endocrine therapy itself or it could be due to the nature of HR + endocrine sensitive disease.

Survival did not change over the study period. Five cases identified in the first two years of the study survived longer than two years, but survival over the decade remained static after accounting for these outliers. Median survival after the diagnosis of BC-LMD was 4.7 months in this study. TNBC LMD had the shortest median survival of 2 months, followed by 5.3 months in HR + and 8.4 months in HER2+. These findings concur with previous studies and BC-LMD survival depending on BC subtype [26, 28]. While longer survival for patients with HER2 + BC-LMD likely stems from HER2 targeted systemic and IT chemotherapy [1, 21, 26, 29], differences in survival in HR + or TNBC may be driven by other factors. Patients with HR + BC-LMD were five times as likely to receive systemic therapy and four times as likely to receive IT chemotherapy following BC-LMD diagnosis compared to those with TNBC. Therefore, intention to treat may be a primary driver of prolonged survival, but other factors including poor performance status and extent of extracranial metastatic disease potentially confound this data [28]. Even so, we were unable to identify any other single factor accounting for differences in survival among patients with HR + or TNBC.

The use of systemic therapy and IT chemotherapy was associated with improved survival following the diagnosis of LMD regardless of HR subtype. IT trastuzumab was associated with prolonged survival in HER2 + LMD [26, 30, 31]. In 2018, the first phase 1 study of IT trastuzumab showed 150 mg weekly dosing achieved steady-state levels after 1 week and was well tolerated [32]. Prior to this publication, patients with HER2 + LMD treated at our institution received weekly IT doses of trastuzumab of less than 150 mg. Our study supports the idea that IT trastuzumab dosing (i.e. physiologic dosing) may result in improved survival for patients with HER2 + disease.

To maximize IT HER2 targeted therapy, our institution opened a phase I/II study of radiotherapy followed by IT trastuzumab and pertuzumab in patients with HER2 + BC-LMD to evaluate safety and treatment outcomes (NCT04588545) [29]. Radiotherapy can eliminate tumor blockages within the leptomeninges, allowing for IT therapy to properly flow along the CSF [33]. The current study, however, found no statistical difference in the OS between patients who received only IT therapy versus WBRT and IT therapy. However, the latter cohort included only four patients, and further investigation is warranted.

There is an increasing interest in HER2-targeting therapies in BC-LMD. Prior studies showed that pertuzumab-based therapies improved progression-free survival (PFS) when used with trastuzumab and taxanes, and when given as a first or second-line chemotherapy [34–36]. Of particular interest in the current study, ten HER2 + BC-LMD patients exhibited a significant increase in OS associated with systemic treatment with lapatinib, a reversible tyrosine kinase inhibitor (TKI) [37, 38]. Previous studies in non-LMD HER2 + advanced BC patients showed that lapatinib with capecitabine is well tolerated [39] and improved PFS to 8.4 months versus 4.4 months using capecitabine alone [40]. Lapatinib + capecitabine BC brain metastases showed overall response rates (ORR) of 59.1% among treatment naïve patients [41] and 21% among patients who may or may not have been exposed to either agent previously [42]. However, the CEREBEL trial demonstrated no difference in PFS between capecitabine plus either lapatinib or trastuzumab [43].The EMILIA trial showed that lapatinib plus capecitabine was both less tolerable and less efficacious than trastuzumab emtansine in prolonging PFS [44]. The LANTERN trial, a phase II trial comparing lapatinib-capecitabine versus trastuzumab-capecitabine therapy in HER2 + BC with CNS metastasis showed no significant difference in PFS but a trend favoring trastuzumab-capecitabine [45]. Aside from two case studies, there is no prospective data describing the efficacy of lapatinib in the treatment of BC-LMD [46, 47].

Neratinib and pyrotinib are similar to lapatinib except that they irreversibly bind to the HER intracellular phosphorylase domain and have efficacy in BC brain metastases [38]. The NALA trial compared lapatinib and neratinib and both with capecitabine in BC brain metastases and showed similar ORR (26.7% versus 32.8%, respectively), but significantly longer duration of response for neratinib rather than lapatinib (8.5 vs. 5.6 months, respectively). The benefits of these TKIs were overshadowed by the combination of T-DM1 and tucatinib, as tolerability was better and efficacy was at least as good for these agents [16, 38]. Neratinib + capecitabine enhanced OS to 10 months and improved neurological symptoms in 60% of patients with HER2 + BC LMD [48]. Tucatinib is a newer TKI that has shown activity in HER2 + BC brain metastases, when combined with trastuzumab and capecitabine [49–51]. CSF pharmacokinetic analysis revealed detectable levels of tucatinib within 2 h of administration (NCT03501979) [52]. Further studies using TKIs in BC-LMD are needed. Other studies evaluated these agents in CNS metastases and the ORR in BC-LMD was not established.

At the present time, there is a lack of effective treatments in TNBC-LMD. The current study showed an association between IT therapy and OS among BC-LMD patients, supporting prior studies [26]. Similarly, we found that patients with TNBC-LMD were less likely to receive treatment after their LMD diagnosis. Prospective trials specifically targeting TNBC-related LMD are critically needed.

A general observation based on the current data conforms with previous work showing that treatment of any type following diagnosis of BC-LMD improved survival to 6.54 months [3, 26, 28]. It might be surmised that greater intention to treat would improve survival, as this study found that patients that did not receive any treatment survived a median of 1.07 months. It is often the case that patients who are eligible to receive these treatments are initially well enough to tolerate them compared to those not receiving treatment, limiting the strength of a median survival comparison between these patient groups. Survival has also been shown to vary when considering diagnostic modality: cytology versus MRI alone [2]. However, we found no difference in OS based on the diagnostic modality. This contradiction may relate to our exclusion of cases of BC-LMD, which were treated on clinical suspicion of LMD but for which no CSF cytology or MRI evidence of disease was found [21]. Furthermore, no differences in the proportion of patients diagnosed by each individual diagnostic modality was observed between 2011 and 2015 (97% MRI; 3% CSF; 28% Both) vs 2016–2020 (96% MRI; 4% CSF; 48% Both) with a greater number of patients receiving both MRI and CSF cytology in the latter half of the decade. The lack of novel, more effective diagnostic modalities in determining LMD is a prominent clinical barrier, accentuating the need for superior approaches to identify disease presence. There may also be improving awareness and recognition of typical radiographic LMD features, which increases the sensitivity of MRI in our institution.

To expand systemic treatment affecting CNS and LMD-related cancer, various novel immunotherapy approaches are being assessed in the management of BC-LMD. The use of systemic pembrolizumab [53] or systemic ipilimumab and nivolumab [54] showed promise for a variety of LMD patients (most of whom had LMD from BC), but the median survival was only 3.6 months and 2.9 months, respectively. Only 11 patients at our institution received systemic immunotherapy following diagnosis with BC-LMD, and no associated benefit was found. An alternative approach in trials now includes IT bispecific antibody-armed T-cells that can be directed against HER2 (NCT03661424). A similar trial with IT HER2-directed chimeric antigen receptor (CAR) T cells (NCT03696030) is currently recruiting. A phase 3 study is planned to use the systemic administration of blood-CSF penetrant drug ANG1005 (a paclitaxel-peptide conjugate that crosses the blood CSF barrier via a low-density lipoprotein receptor-related protein-1 (LRP-1) mediated transcytosis). A phase 2 study with the same agent showed activity and an average survival of 8 months in BC patients with LMD [55]. At the time of this report, there are only 23 recruiting or active clinical trials in the US targeting LMD generally and only 12 targeting BC-LMD which are summarized in Additional file 1: Table S3. Even with observed improvements in outcomes for HER2 + BC-LMD, the need to find new therapies which improve OS in BC-LMD is dire. Overall, while this data is limited to a single center, retrospectively analyzed experience here at MCC, the reported outcomes of this study provide an exploratory report of BC LMD cases which can be used to inform clinical decisions and influence the direction of future research.

### Supplementary Information

**Additional file 1: Supplementary Table 1**. Univariable Cox proportional hazard regression analysis to estimate Hazard Ratios (HR) and 95% confidence intervals (CI) for various treatments administered to patients post CNS metastasis/pre BC-LMD diagnosis. Treatments that were found to significantly prolong time between CNS metastasis and BC-LMD are highlighted in red. ** Supplementary Table 2**. Univariable Cox proportional hazard regression analysis to estimate Hazard Ratios (HR) and 95% confidence intervals (CI) for various treatments administered to patients post BC-LMD diagnosis. Treatments found to significantly enhance overall survival are highlighted in red. Abbreviations: Hormone Receptor Positive (HR+); Human Epidermal Growth Factor Receptor 2-Positive (HER2+); Triple Negative Breast Cancer (TNBC); Immune Checkpoint Inhibitors (ICI); Intrathecal Therapy (IT); Systemic (sys); Whole Brain Radiation Therapy (WBRT); Hazard Ratio (HR); Confidence Interval (CI).** Supplementary Table 3**. Actively recruiting or upcoming but not yet recruiting clinical trials including breast cancer patients with metastasis to the leptomeninges. Abbreviations: Leptomeningeal Disease (LMD); Radiotherapy (RT) or (XRT); Whole Brain Radiation Therapy (WBRT); Hormone Receptor Positive (HR+); Human Epidermal Growth Factor Receptor 2-Positive (HER2+); Intrathecal Therapy (IT).

**Additional file 2: Supplementary Figure 1**. Kaplan-Meier estimates and multivariate analyses for treatments that affect time between CNS-metastasis diagnosis and BC-LMD diagnosis in HR+ BC patients. A) HR+ patients that received any hormone therapy experienced a significantly longer median time between breast cancer CNS-metastasis diagnosis and BC-LMD (25.6 months) compared to patients that did not (4.6 months). In particular, HR+ patients receiving the hormone therapy letrozole (B) had a longer median time between CNS-metastasis and BC-LMD compared to patients that did not (27.3 months vs 4.9 months, respectively). Patients receiving hormone therapy exemestane (C) also experienced longer times between CNS metastasis and LMD (27.8 months) compared to those patients that did not (6.1 months). D) HR+ patients receiving CDK4 and CDK6 selective inhibitor Palbociclib post CNS-metastasis experienced longer times between CNS metastasis and BC-LMD diagnoses compared to those that were not treated with Palbociclib (26.8 vs 7.2 months). E) HR+ patients receiving kinase inhibitor Everolimus experienced significantly longer times between CNS metastasis and BC-LMD (27.6 months) compared to those that did not (7.2 months). F) Multivariate analysis demonstrates that exemestane and Palbociclib are significant in delaying progression from CNS metastasis to BC-LMD in HR+ patients. Abbreviations: Leptomeningeal Disease (LMD); Hazard Ratio (HR); Confidence Interval (CI).** Supplementary Figure 2**. Kaplan-Meier estimate for the efficacy of systemic lapatinib in delaying progression of CNS metastasis to BC-LMD in HER2+ positive BC-patients. HER2+ Patients receiving systemic lapatinib post CNS metastasis diagnosis had a median time of 20.9 months before BC-LMD diagnosis, compared to a median time of 5.7 months for patients of any BC subtype who did not receive systemic lapatinib. Abbreviations: Breast Cancer (BC); Central Nervous System (CNS); Leptomeningeal Disease (LMD).** Supplementary Figure 3**. Kaplan-Meier estimate for surgery and radiosurgery affecting time between BC CNS metastasis and BC LMD. Patients who underwent tumor resection (**A**) or radiosurgery (**B**) did not experience differences in time between CNS metastasis and LMD when compared to patients who did not undergo these respective treatments. Abbreviations: Breast Cancer (BC); Central Nervous System (CNS); Leptomeningeal Disease (LMD).** Supplementary Figure 4**. Kaplan-Meier estimate for treatments affecting overall survival in HR+ BC-LMD patients. Patients receiving intrathecal therapy (A) had a significantly higher median overall survival time (9.9 months) when compared to HR+ patients that did not receive any intrathecal therapy (2.6 months). More specifically, HR+ patients receiving intrathecal methotrexate (B) had a higher median overall survival time (9.5 months) compared to those that did not (2.9 months). Abbreviations: Hormone Receptor Positive (HR+); Breast Cancer Leptomeningeal Disease (BC-LMD).** Supplementary Figure 5**. Kaplan-Meier estimate for treatments affecting overall survival in HER2+ BC-LMD patients post BC-LMD diagnosis. A) HER2+ patients receiving systemic therapy had a higher median overall survival time (12 months) compared to those that did not receive any systemic treatment (1.9 months). B-C) Specifically, overall median survival time was higher in patients receiving systemic trastuzumab (12.7 months) and/or systemic lapatinib (9.8 months) when compared to patients that did not (7.2 months; 7.4 months, respectively). D) HER2+ BC-LMD patients receiving intrathecal therapy had a higher median overall survival time (12.6 months) than those that did not (1.5 months). All HER2+ patients receiving IT therapy received IT trastuzumab. Abbreviations: Human Epidermal Growth Factor Receptor 2-Positive (HER2+); Breast Cancer Leptomeningeal Disease (BC-LMD).** Supplementary Figure 6**. Kaplan-Meier estimate for treatments affecting overall survival in TNBC-LMD patients post BC-LMD diagnosis. A) TNBC-LMD patients receiving systemic therapy had a higher median overall survival time (3.4 months) compared to those that did not receive any systemic treatment (1.2 months). B) TNBC-LMD patients receiving intrathecal therapy had a higher median overall survival time (8.9 months) than those that did not (1.1 months). Abbreviations: Triple Negative Breast Cancer (TNBC); Breast Cancer Leptomeningeal Disease (BC-LMD).

## Data Availability

All data and materials are stored in a safe, protected drive that is available for review.
