# Supplementary material for: Factors associated with overall survival in breast cancer patients with leptomeningeal disease (LMD): a single institutional retrospective review
Source: Breast Cancer Res. 2024 Mar 29;26:55. doi: 10.1186/s13058-024-01789-7 (PMC10979566; doi:10.1186/s13058-024-01789-7)
Supplement: Supplementary file 1 — Additional file 1: Supplementary Table 1. Univariable Cox proportional hazard regression analysis to estimate Hazard Ratios (HR) and 95% confidence intervals (CI) for various treatments administered to patients post CNS metastasis/pre BC-LMD diagnosis. Treatments that were found to significantly prolong time between CNS metastasis and BC-LMD are highlighted in red. Supplementary Table 2. Univariable Cox proportional hazard regression analysis to estimate Hazard Ratios (HR) and 95% confidence intervals (CI) for various treatments administered to patients post BC-LMD diagnosis. Treatments found to significantly enhance overall survival are highlighted in red. Abbreviations: Hormone Receptor Positive (HR+); Human Epidermal Growth Factor Receptor 2-Positive (HER2+); Triple Negative Breast Cancer (TNBC); Immune Checkpoint Inhibitors (ICI); Intrathecal Therapy (IT); Systemic (sys); Whole Brain Radiation Therapy (WBRT); Hazard Ratio (HR); Confidence Interval (CI). Supplementary Table 3. Actively recruiting or upcoming but not yet recruiting clinical trials including breast cancer patients with metastasis to the leptomeninges. Abbreviations: Leptomeningeal Disease (LMD); Radiotherapy (RT) or (XRT); Whole Brain Radiation Therapy (WBRT); Hormone Receptor Positive (HR+); Human Epidermal Growth Factor Receptor 2-Positive (HER2+); Intrathecal Therapy (IT). [file 13058_2024_1789_MOESM1_ESM.docx]

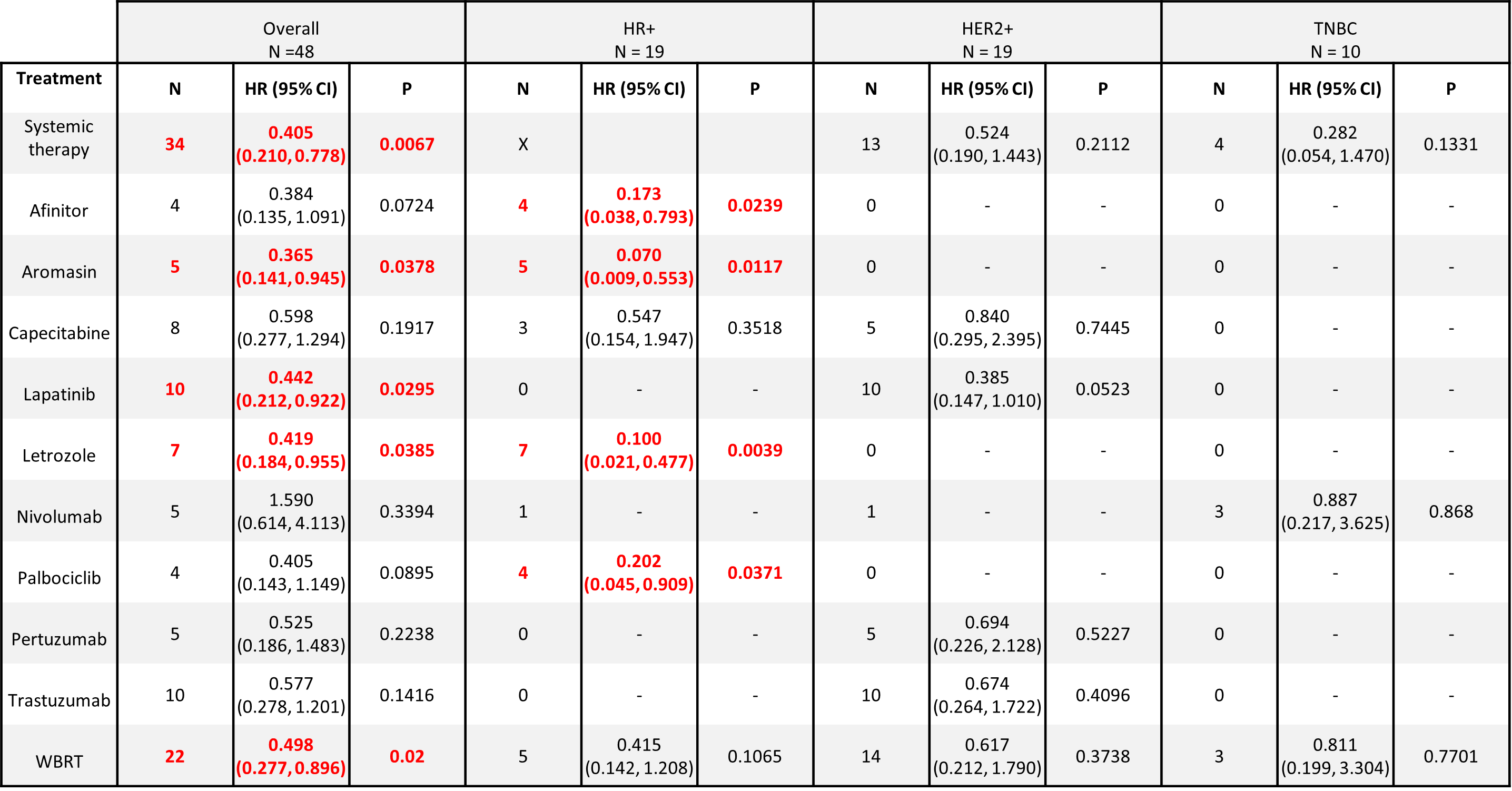


| **Supplementary Table 1.** Univariable Cox proportional hazard regression analysis to estimate Hazard Ratios (HR) and 95% confidence intervals (CI) for various treatments administered to patients post CNS metastasis/pre BC-LMD diagnosis. Treatments that were found to significantly prolong time between CNS metastasis and BC-LMD are highlighted in red. |
| --- |
| **Abbreviations:** Hormone Receptor Positive (HR+); Human Epidermal Growth Factor Receptor 2-Positive (HER2+); Triple Negative Breast Cancer (TNBC); Whole Brain Radiation Therapy (WBRT); Hazard Ratio (HR); Confidence Interval (CI). |


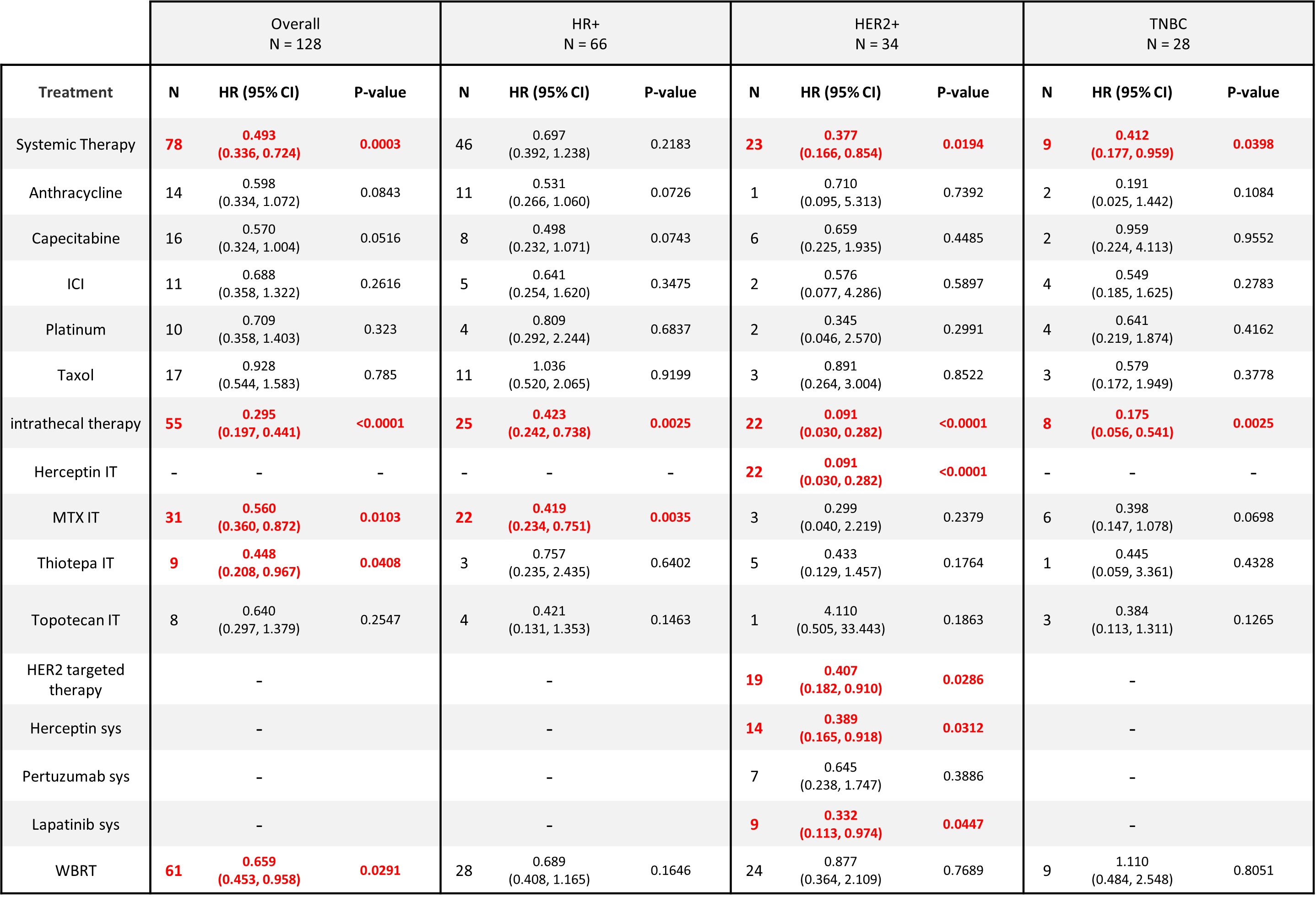


| **Supplementary Table 2.** Univariable Cox proportional hazard regression analysis to estimate Hazard Ratios (HR) and 95% confidence intervals (CI) for various treatments administered to patients post BC-LMD diagnosis. Treatments found to significantly enhance overall survival are highlighted in red. |
| --- |
| **Abbreviations:** Hormone Receptor Positive (HR+); Human Epidermal Growth Factor Receptor 2-Positive (HER2+); Triple Negative Breast Cancer (TNBC); Immune Checkpoint Inhibitors (ICI); Intrathecal Therapy (IT); Systemic (sys); Whole Brain Radiation Therapy (WBRT); Hazard Ratio (HR); Confidence Interval (CI). |


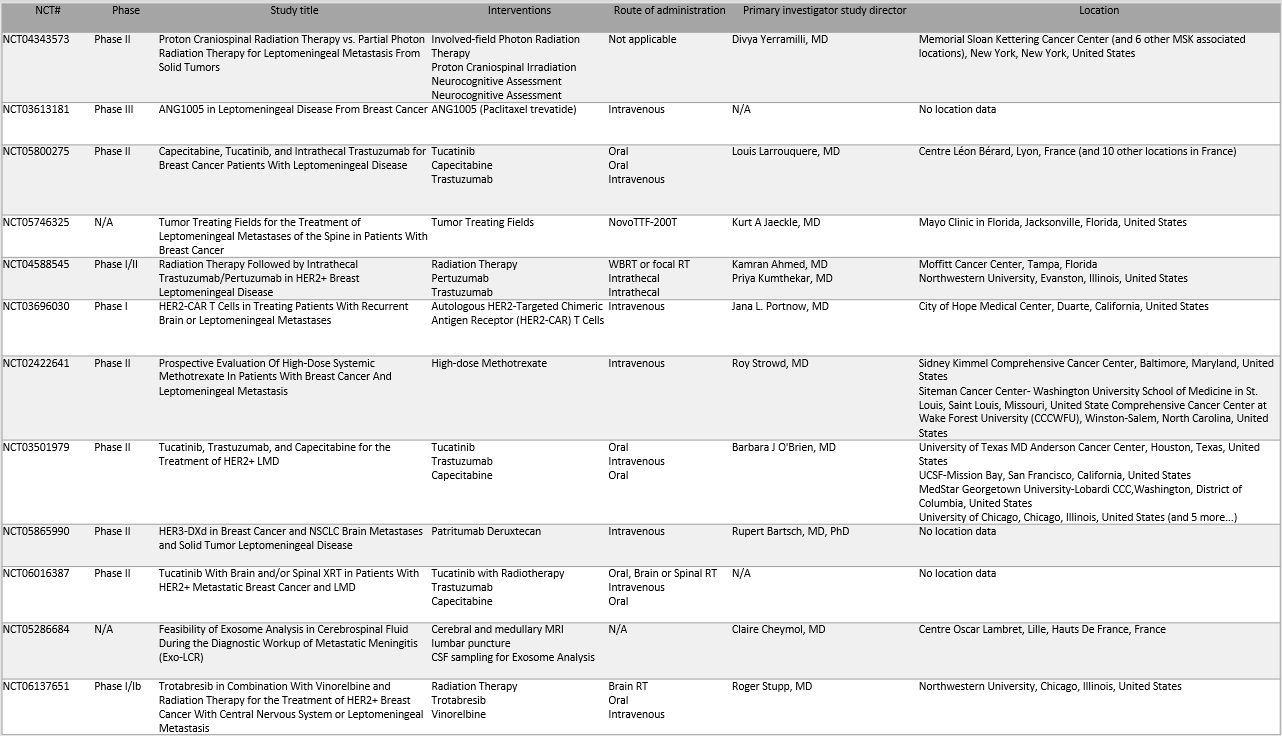


| **Supplementary Table 3.** Actively recruiting or upcoming but not yet recruiting clinical trials including breast cancer patients with metastasis to the leptomeninges. |
| --- |
| **Abbreviations:** Leptomeningeal Disease (LMD); Radiotherapy (RT) or (XRT); Whole Brain Radiation Therapy (WBRT); Hormone Receptor Positive (HR+); Human Epidermal Growth Factor Receptor 2-Positive (HER2+); Intrathecal Therapy (IT). |
