# Supplementary material for: Factors associated with overall survival in breast cancer patients with leptomeningeal disease (LMD): a single institutional retrospective review
Source: Breast Cancer Res. 2024 Mar 29;26:55. doi: 10.1186/s13058-024-01789-7 (PMC10979566; doi:10.1186/s13058-024-01789-7)
Supplement: Supplementary file 2 — Additional file 2: Supplementary Figure 1. Kaplan-Meier estimates and multivariate analyses for treatments that affect time between CNS-metastasis diagnosis and BC-LMD diagnosis in HR+ BC patients. A) HR+ patients that received any hormone therapy experienced a significantly longer median time between breast cancer CNS-metastasis diagnosis and BC-LMD (25.6 months) compared to patients that did not (4.6 months). In particular, HR+ patients receiving the hormone therapy letrozole (B) had a longer median time between CNS-metastasis and BC-LMD compared to patients that did not (27.3 months vs 4.9 months, respectively). Patients receiving hormone therapy exemestane (C) also experienced longer times between CNS metastasis and LMD (27.8 months) compared to those patients that did not (6.1 months). D) HR+ patients receiving CDK4 and CDK6 selective inhibitor Palbociclib post CNS-metastasis experienced longer times between CNS metastasis and BC-LMD diagnoses compared to those that were not treated with Palbociclib (26.8 vs 7.2 months). E) HR+ patients receiving kinase inhibitor Everolimus experienced significantly longer times between CNS metastasis and BC-LMD (27.6 months) compared to those that did not (7.2 months). F) Multivariate analysis demonstrates that exemestane and Palbociclib are significant in delaying progression from CNS metastasis to BC-LMD in HR+ patients. Abbreviations: Leptomeningeal Disease (LMD); Hazard Ratio (HR); Confidence Interval (CI). Supplementary Figure 2. Kaplan-Meier estimate for the efficacy of systemic lapatinib in delaying progression of CNS metastasis to BC-LMD in HER2+ positive BC-patients. HER2+ Patients receiving systemic lapatinib post CNS metastasis diagnosis had a median time of 20.9 months before BC-LMD diagnosis, compared to a median time of 5.7 months for patients of any BC subtype who did not receive systemic lapatinib. Abbreviations: Breast Cancer (BC); Central Nervous System (CNS); Leptomeningeal Disease (LMD). Supplementary Figur [file 13058_2024_1789_MOESM2_ESM.docx]

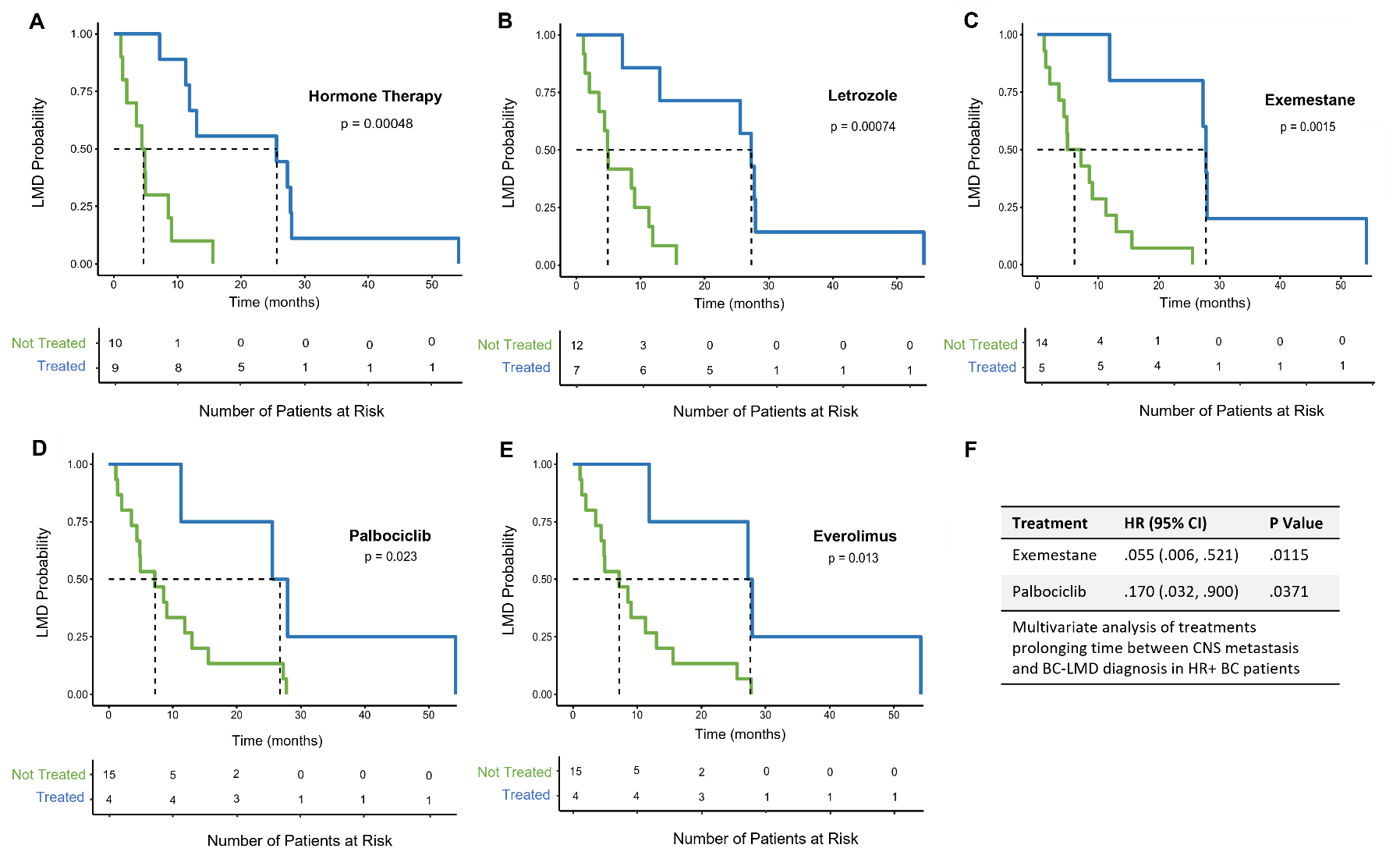


| **Supplementary Figure 1.** Kaplan-Meier estimates and multivariate analyses for treatments that affect time between CNS-metastasis diagnosis and BC-LMD diagnosis in HR+ BC patients. **A)** HR+ patients that received any hormone therapy experienced a significantly longer median time between breast cancer CNS-metastasis diagnosis and BC-LMD (25.6 months) compared to patients that did not (4.6 months). In particular, HR+ patients receiving the hormone therapy letrozole **(B)** had a longer median time between CNS-metastasis and BC-LMD compared to patients that did not (27.3 months vs 4.9 months, respectively). Patients receiving hormone therapy exemestane **(C)** also experienced longer times between CNS metastasis and LMD (27.8 months) compared to those patients that did not (6.1 months). **D)** HR+ patients receiving CDK4 and CDK6 selective inhibitor Palbociclib post CNS-metastasis experienced longer times between CNS metastasis and BC-LMD diagnoses compared to those that were not treated with Palbociclib (26.8 vs 7.2 months). **E)** HR+ patients receiving kinase inhibitor Everolimus experienced significantly longer times between CNS metastasis and BC-LMD (27.6 months) compared to those that did not (7.2 months). **F)** Multivariate analysis demonstrates that exemestane and Palbociclib are significant in delaying progression from CNS metastasis to BC-LMD in HR+ patients. |
| --- |
| **Abbreviations:** Leptomeningeal Disease (LMD); Hazard Ratio (HR); Confidence Interval (CI). |


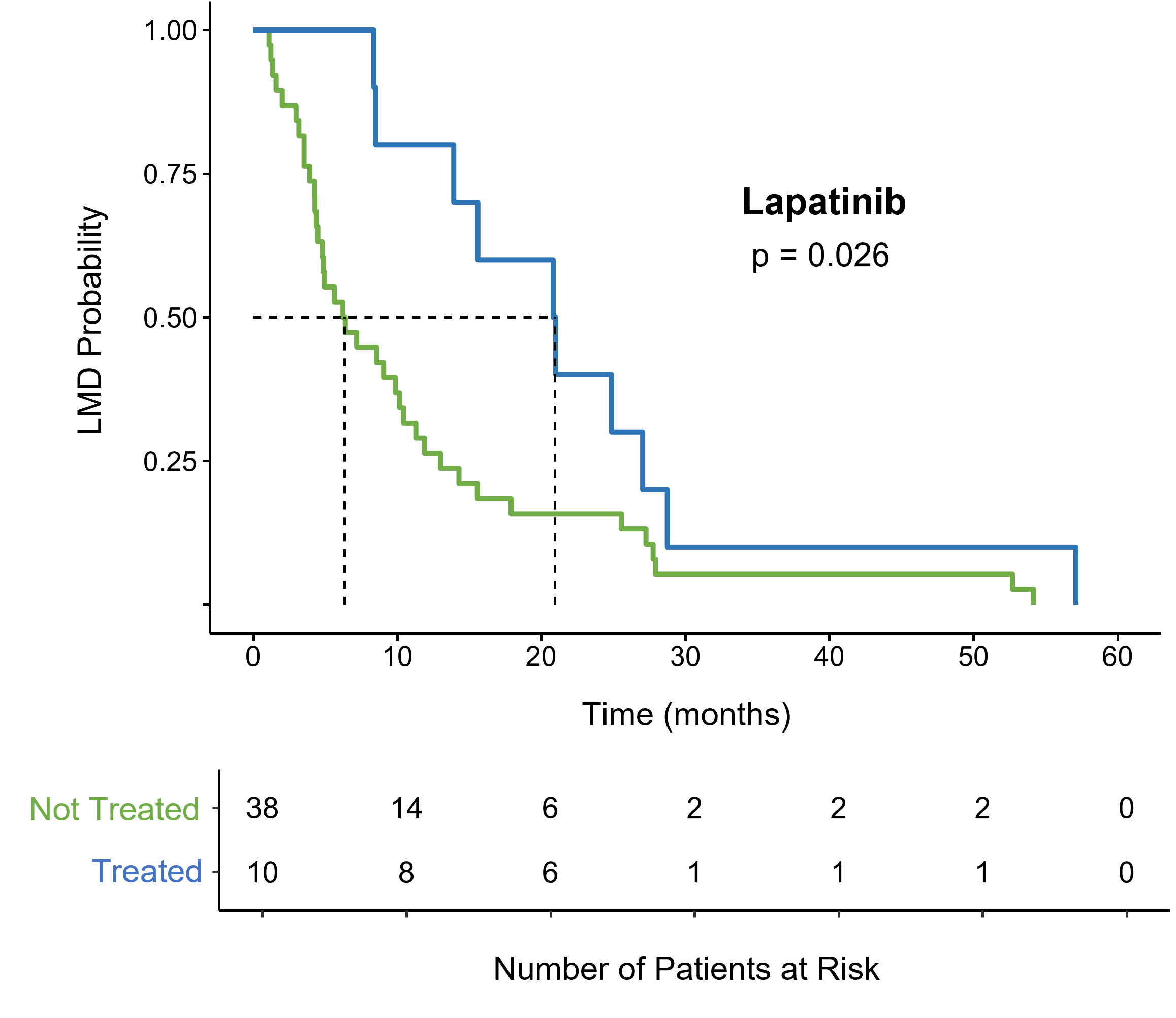


| **Supplementary Figure 2.** Kaplan-Meier estimate for the efficacy of systemic lapatinib in delaying progression of CNS metastasis to BC-LMD in HER2+ positive BC-patients. HER2+ Patients receiving systemic lapatinib post CNS metastasis diagnosis had a median time of 20.9 months before BC-LMD diagnosis, compared to a median time of 5.7 months for patients of any BC subtype who did not receive systemic lapatinib. |
| --- |
| **Abbreviations:** Breast Cancer (BC); Central Nervous System (CNS); Leptomeningeal Disease (LMD) |


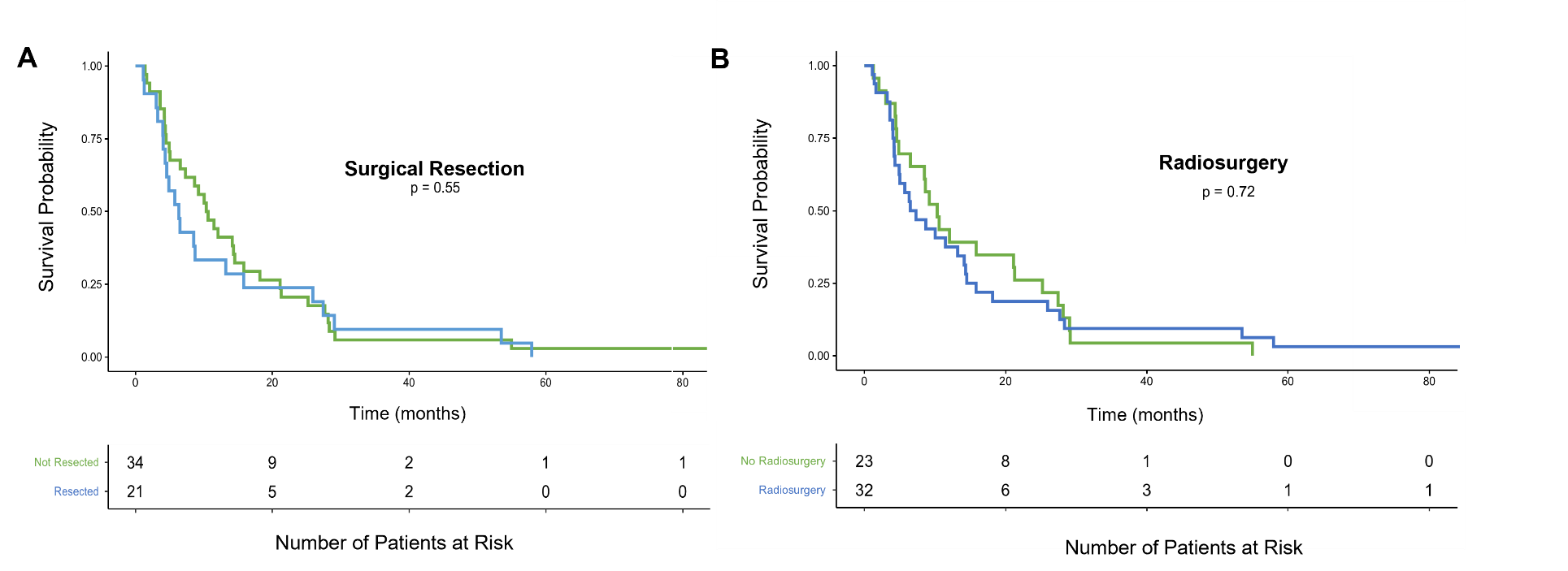


| **Supplementary Figure 3.** Kaplan-Meier estimate for surgery and radiosurgery affecting time between BC CNS metastasis and BC LMD. Patients who underwent tumor resection **(A)** or radiosurgery **(B)** did not experience differences in time between CNS metastasis and LMD when compared to patients who did not undergo these respective treatments. |
| --- |
| **Abbreviations:** Breast Cancer (BC); Central Nervous System (CNS); Leptomeningeal Disease (LMD). |


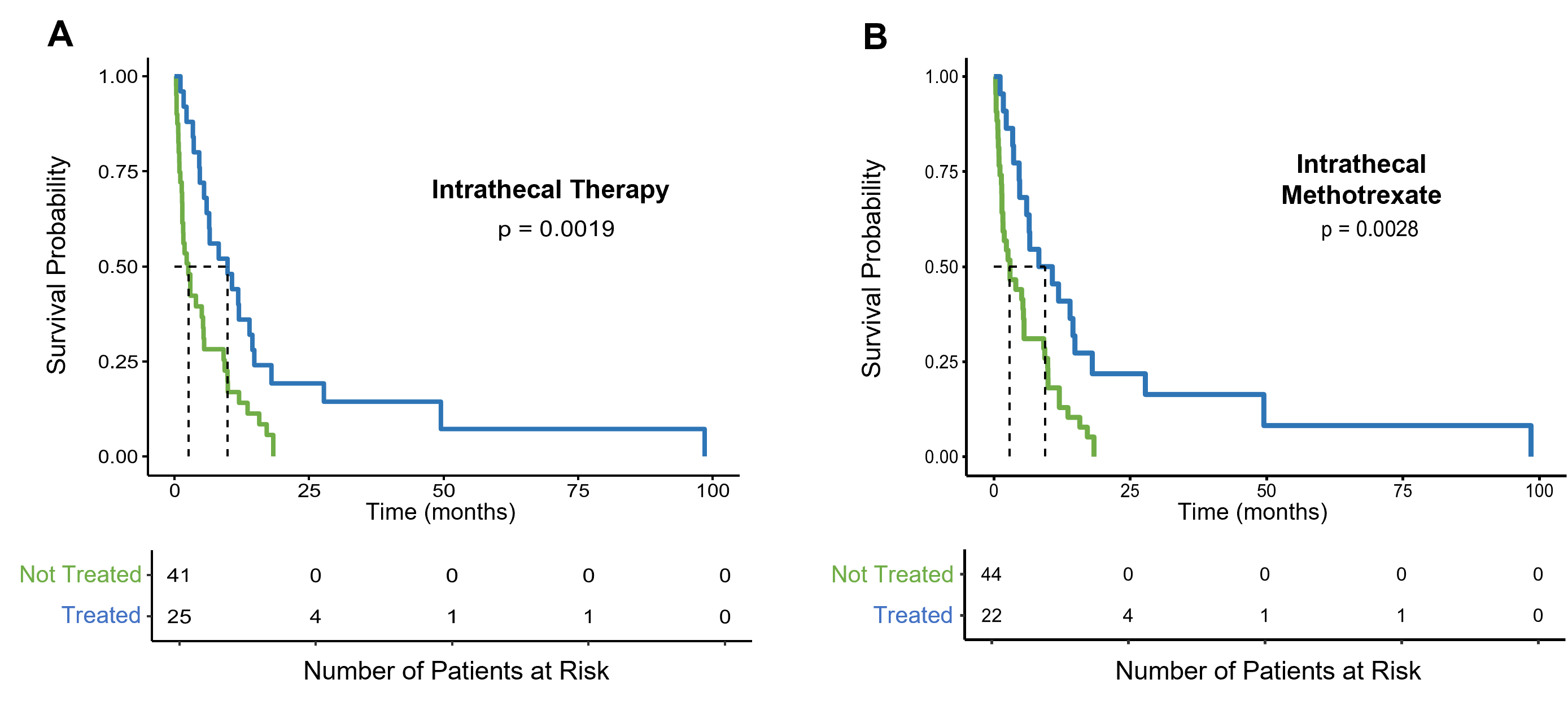


| **Supplementary Figure 4.** Kaplan-Meier estimate for treatments affecting overall survival in HR+ BC-LMD patients. Patients receiving intrathecal therapy **(A)** had a significantly higher median overall survival time (9.9 months) when compared to HR+ patients that did not receive any intrathecal therapy (2.6 months). More specifically, HR+ patients receiving intrathecal methotrexate **(B)** had a higher median overall survival time (9.5 months) compared to those that did not (2.9 months). |
| --- |
| **Abbreviations:** Hormone Receptor Positive (HR+); Breast Cancer Leptomeningeal Disease (BC-LMD). |


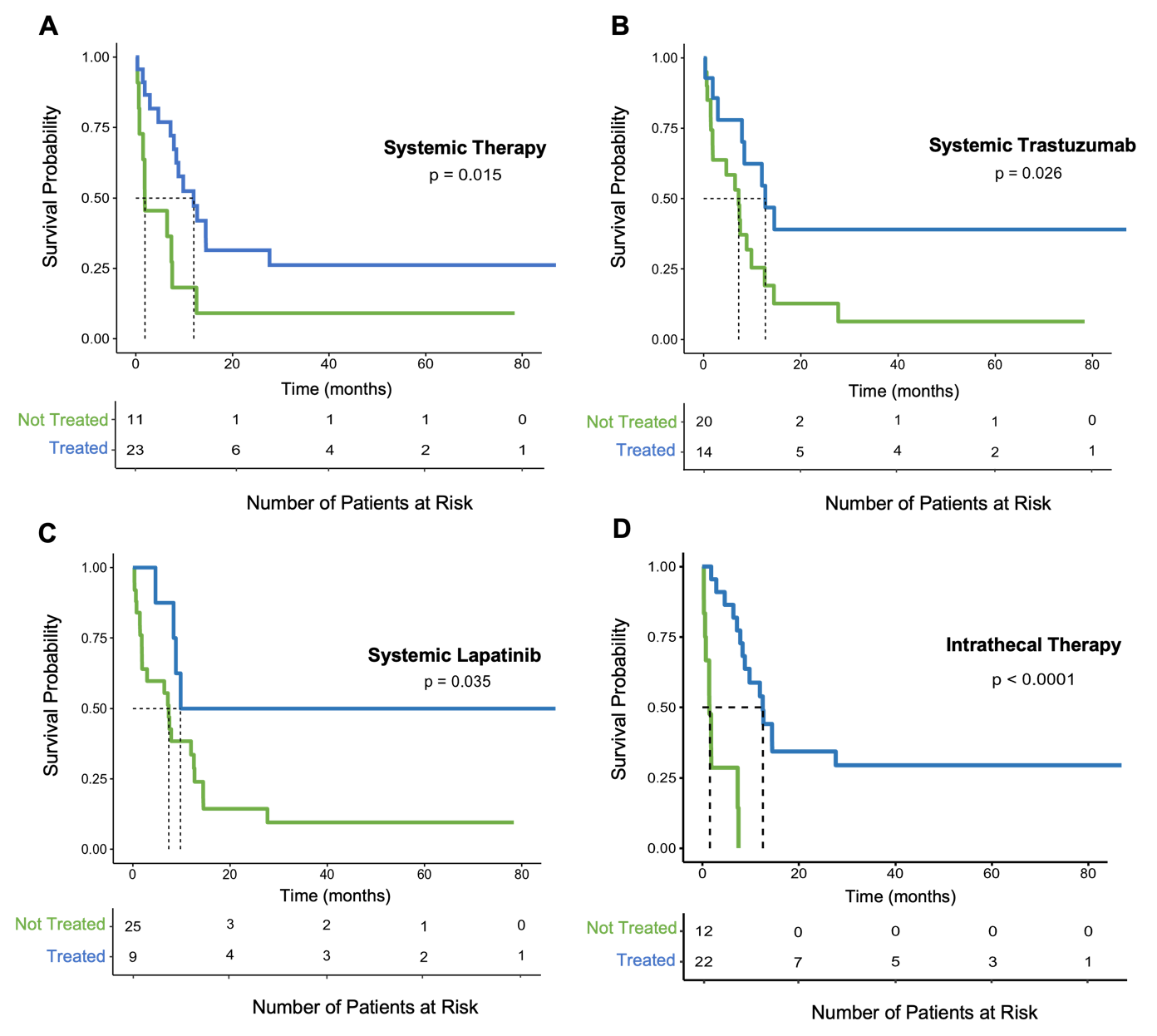


| **Supplementary Figure 5.** Kaplan-Meier estimate for treatments affecting overall survival in HER2+ BC-LMD patients post BC-LMD diagnosis. **A)** HER2+ patients receiving systemic therapy had a higher median overall survival time (12 months) compared to those that did not receive any systemic treatment (1.9 months). **B-C)** Specifically, overall median survival time was higher in patients receiving systemic trastuzumab (12.7 months) and/or systemic lapatinib (9.8 months) when compared to patients that did not (7.2 months; 7.4 months, respectively). **D)** HER2+ BC-LMD patients receiving intrathecal therapy had a higher median overall survival time (12.6 months) than those that did not (1.5 months). All HER2+ patients receiving IT therapy received IT trastuzumab. |
| --- |
| **Abbreviations:** Human Epidermal Growth Factor Receptor 2-Positive (HER2+); Breast Cancer Leptomeningeal Disease (BC-LMD). |


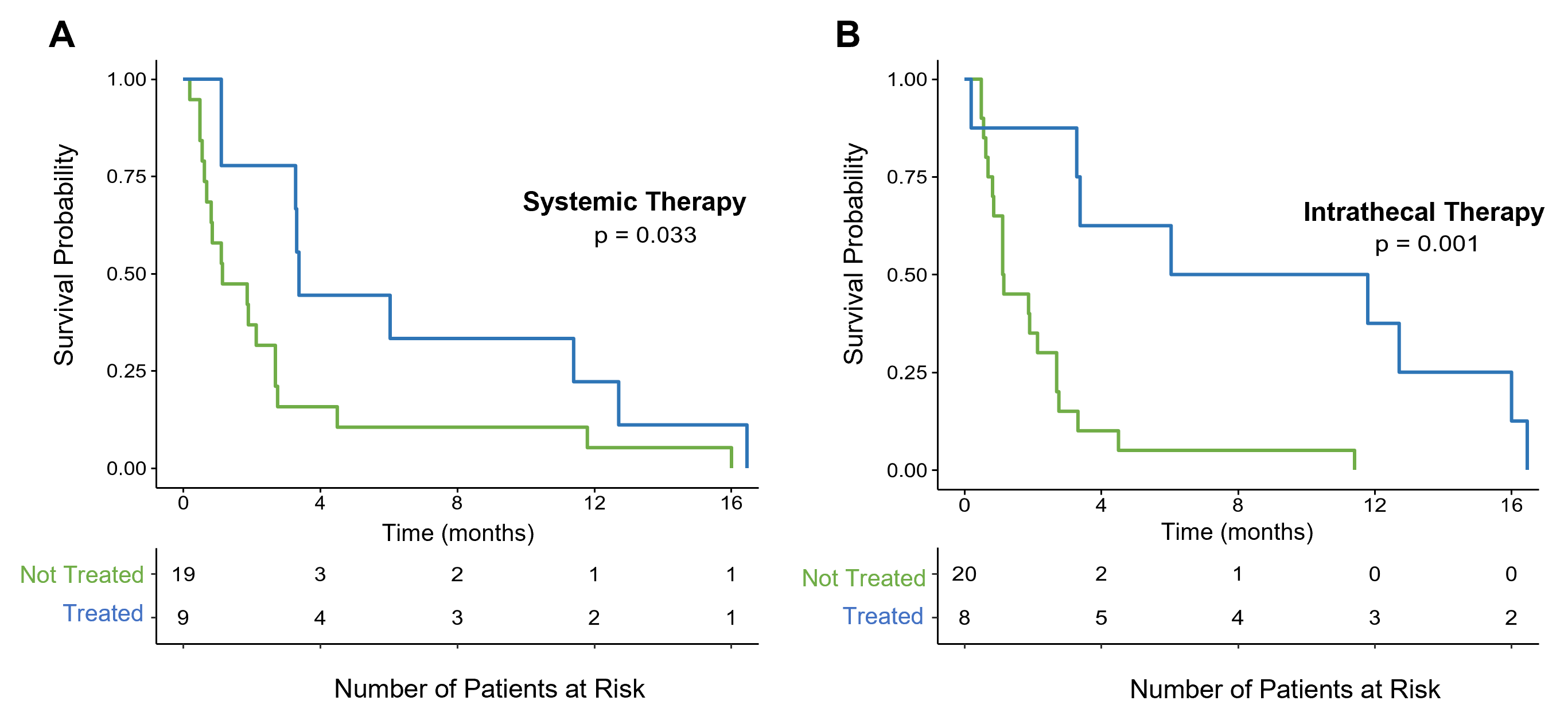


| **Supplementary Figure 6.** Kaplan-Meier estimate for treatments affecting overall survival in TNBC-LMD patients post BC-LMD diagnosis. **A)** TNBC-LMD patients receiving systemic therapy had a higher median overall survival time (3.4 months) compared to those that did not receive any systemic treatment (1.2 months). **B)** TNBC-LMD patients receiving intrathecal therapy had a higher median overall survival time (8.9 months) than those that did not (1.1 months). |
| --- |
| **Abbreviations:** Triple Negative Breast Cancer (TNBC); Breast Cancer Leptomeningeal Disease (BC-LMD). |
